# In-office dispensing of oral targeted agents by urology practices in men with advanced prostate cancer

**DOI:** 10.1093/jncics/pkad062

**Published:** 2023-08-29

**Authors:** Dawson Hill, Samuel R Kaufman, Mary K Oerline, Kassem Faraj, Megan E V Caram, Vahakn B Shahinian, Brent K Hollenbeck, Avinash Maganty

**Affiliations:** Dow Division of Health Services Research, Department of Urology, University of Michigan, Ann Arbor, MI, USA; Dow Division of Health Services Research, Department of Urology, University of Michigan, Ann Arbor, MI, USA; Dow Division of Health Services Research, Department of Urology, University of Michigan, Ann Arbor, MI, USA; Dow Division of Health Services Research, Department of Urology, University of Michigan, Ann Arbor, MI, USA; Division of Hematology/Oncology, Department of Internal Medicine, University of Michigan, Ann Arbor, MI, USA; Veterans Affairs (VA) Health Services Research & Development, Center for Clinical Management Research, VA Ann Arbor Healthcare System, Ann Arbor, MI, USA; Dow Division of Health Services Research, Department of Urology, University of Michigan, Ann Arbor, MI, USA; Division of Nephrology, Department of Internal Medicine, University of Michigan, Ann Arbor, MI, USA; Dow Division of Health Services Research, Department of Urology, University of Michigan, Ann Arbor, MI, USA; Dow Division of Health Services Research, Department of Urology, University of Michigan, Ann Arbor, MI, USA

## Abstract

**Background:**

Management of men with advanced prostate cancer has evolved to include urologists, made possible by oral targeted agents (eg, abiraterone or enzalutamide) that can be dispensed directly to patients in the office. We sought to investigate whether this increasingly common model improves access to these agents, especially for Black men who are historically undertreated.

**Methods:**

We used 20% national Medicare data to perform a retrospective cohort study of men with advanced prostate cancer from 2011 through 2019, managed by urology practices with and without in-office dispensing. Using a difference-in-difference framework, generalized estimating equations were used to measure the effect of in-office dispensing on prescriptions for abiraterone and/or enzalutamide, adjusting for differences between patients, including race.

**Results:**

New prescription fills for oral targeted agents increased after the adoption of in-office dispensing (+4.4%, 95% confidence interval [CI] = 3.4% to 5.4%) relative to that for men managed by practices without dispensing (+2.4%, 95% CI = 1.4% to 3.4%). The increase in the postintervention period (difference-in-difference estimate) was 2% higher (95% CI = 0.6% to 3.4%) for men managed by practices adopting dispensing relative to men managed by practices without dispensing. The effect was strongest for practices adopting dispensing in 2015 (difference-in-difference estimate: +4.2%, 95% CI = 2.3% to 6.2%). The effect of dispensing adoption did not differ by race.

**Conclusion:**

Adoption of in-office dispensing by urology practices increased prescription fills for oral targeted agents in men with advanced prostate cancer. This model of delivery may improve access to this important class of medications.

Cytotoxic chemotherapy, such as docetaxel, is used to treat men with advanced prostate cancer. However, with the approval of abiraterone in 2011, the therapeutic landscape has evolved considerably (1). Although not necessarily a substitution for chemotherapy, the increasing availability of targeted agents for prostate cancer has the potential to reduce treatment burden because of their improved toxicity profiles and the convenience of oral administration. Nonetheless, access to these medications is not ubiquitous given highly variable prescribing practices that are independent of patient clinical characteristics (2). Moreover, men with prostate cancer often experience difficulty obtaining these medications because of associated high out-of-pocket costs (3).

There has been a growing trend in urology to provide medications through in-office dispensing (4,5). However, the effect of this form of care delivery on access to oral targeted agents for advanced prostate cancer remains unclear. On the one hand, potential financial incentives from in-office dispensing, whereby a practice collects a margin on each prescription filled, may encourage prescribing behaviors in those familiar with the medications and/or encourage others to begin prescribing them, both of which can improve access. On the other hand, in-office dispensing may impose additional barriers to prescriptions by charging higher prices or by not offering less expensive alternatives. Importantly, depending on where and how it is deployed, in-office dispensing has the potential to mitigate existing racial disparities in treatment of advanced prostate cancer. For example, despite being more likely to respond to targeted agents, Black men are less likely to receive these medications and more commonly have difficulty adhering to treatment, potentially because of insufficient insurance coverage, cost, and reduced access (3,6-8). As urologist involvement and the availability of in-office dispensing grows, understanding the effects of this delivery model is critical.

For this reason, we performed a national study of Medicare beneficiaries with advanced prostate cancer to evaluate the effect of in-office dispensing on prescriptions for abiraterone and/or enzalutamide. Additionally, we examined how this effect varied between Black and non-Black men. We hypothesized that in-office dispensing would increase prescriptions for oral targeted agents for men with advanced prostate cancer, irrespective of race.

## Methods

### Data sources and study population

We identified men with advanced prostate cancer between 2011 and 2019 in a 20% random sample of fee-for-service Medicare beneficiaries using established methods (9). Briefly, men with advanced prostate cancer were identified as those being treated with chronic androgen deprivation therapy (ie, bilateral orchiectomy or at least 6 months of continuous use of leuprolide, gosereline, degarelix, or triptoerlin). We excluded men receiving androgen deprivation therapy as an adjunct to local treatment or who had undergone local treatment in the 12-month period prior to initiating androgen deprivation therapy for the first time. Men participating in Medicare Advantage were also excluded to ensure the complete availability of claims.

Men with advanced prostate cancer were assigned to their primary cancer specialist, inclusive of urologists or medical oncologists, using established methods based on the plurality of evaluation and management services associated with a diagnosis of prostate cancer (10). Because we were interested in the recent phenomena of in-office dispensing by urology practices, we excluded men whose primary cancer specialist was a medical oncologist. Next, using the Medicare Data on Provider Practice and Specialty file, each urologist was assigned to his or her group practice based on the tax identification number under which the majority of his or her claims were submitted in that calendar year (11). Practices were further characterized by their organizational context (ie, single specialty vs multispecialty groups) using established methods (10,12). We limited the analysis to men managed by single specialty urology practices because of their adoption of in-office dispensing (13). Additionally, because no small practices (1-2 urologists) adopted in-office dispensing, the analysis was further limited to men managed by medium (3 or more urologists) and large (10 or more urologists) single specialty urology practices.

### In-office dispensing

The exposure of interest was adoption of in-office dispensing, which was measured at the practice level. In-office dispensing was identified using data from the National Council for Prescription Drug Programs for each year of the study period (14). The National Council for Prescription Drug Programs is an organization that gathers and validates data on licensed pharmacies across the United States, which is used by Medicare and other federal agencies (14). A practice was considered to provide in-office dispensing if its tax identification number was listed as a dispensing site. Because of the difference-in-difference design (discussed below), only practices present in the 2 years before and after adoption were included. Control urology practices (ie, those that never adopted in-office dispensing) were randomly matched to dispensing practices proportionally based on the year of adoption.

### Prescription fills for oral targeted agents

The outcome of interest, measured at the patient level, was a prescription fill for abiraterone and/or enzalutamide. Prescription fills for abiraterone and/or enzalutamide were measured using the Medicare Part D file.

### Statistical analysis

Bivariate analyses were performed to compare patient characteristics between men managed in practices with and without in-office dispensing. χ^2^ test was used to compare categorical data, and student *t* test was used to compare continuous data.

A difference-in-difference analysis was used to estimate changes in the probability of receiving a new prescription for abiraterone and/or enzalutamide after adoption of in-office dispensing. Difference-in-difference is a quasi-experimental design that allows for estimation of the causal effect of a treatment or policy by accounting for secular trends. This approach requires identification of an exposed and control group before and after policy adoption. First, 2 differences are computed: the difference before and after the policy in the exposed group and the difference before and after the policy in the control group (15). The difference of these differences represents the impact of the policy. The assumptions for this framework that allow for causal inference include parallel trends in the outcome before the intervention in both treated and control groups. We confirmed the trends in prescriptions were parallel for both practices with and without dispensing in the pre-intervention period (*P *=* *.69, for difference in pre-intervention slopes between practices with and without dispensing).

Generalized estimating equations with a logit link and binomial family distribution were used to estimate the dichotomous outcome of whether a patient received a prescription for abiraterone and/or enzalutamide. Variables indicating the exposure (ie, practice with or without dispensing) and the intervention periods (ie, before and after in-office dispensing) were included in the model. The intervention periods were defined relative to the year dispensing was adopted for a given practice (ie, pre-intervention period: years -2 and -1, postintervention period: years +1 and +2). The year dispensing was adopted (year 0) was treated as a lead-in period and not included in the analysis. Control practices without dispensing were assigned the same year 0 as their corresponding matched practice with dispensing. An interaction term between the exposure variable and the intervention period was included, serving as the difference-in-difference estimator. All models were adjusted for patient characteristics including age, comorbid conditions (16), socioeconomic status (17), self-reported race, and months on androgen deprivation therapy. Months on androgen deprivation therapy served as a surrogate for the phase of advanced disease, as those who have been on therapy for a longer period of time are more likely to progress to the castrate-resistant phase for which targeted agents were initially approved (18).

Next, we considered whether the effect of dispensing varied by year of adoption (ie, early adoption vs late adoption), given that the study spanned a period during which indications for oral targeted agents expanded. For instance, in-office dispensing became prevalent among urology practices beginning in 2015 (13), during which time targeted agents were approved for the late phase of advanced prostate cancer (ie, castrate-resistant disease). However, indications for targeted agents expanded to include an earlier phase of advanced disease (ie, castrate sensitive) after clinical trials demonstrated efficacy in 2017 (1). Given these evolving indications for targeted agents, we assessed whether the effect of dispensing varied among practices adopting dispensing earlier in the study period vs later. To do this, rather than aggregate the data into a single model, we estimated separate models for each year in which dispensing was adopted (ie, 2015, 2016, and 2017). We considered the first year of in-office dispensing among urology practices to be 2015, as its prevalence among urology practices prior to this year was low (less than 3%) (19). The last year in-office dispensing was evaluated was 2017 to allow for 2 years of data in the postintervention period (nota bene [NB], data was available through 2019).

Finally, we assessed whether the effect of in-office dispensing differed by race. To do this, we repeated the analyses but additionally added a race interaction (ie, Black vs non-Black men) to the difference-in-difference estimator.

All analyses were performed using SAS version 9.4 (Cary, NC, USA) and Stata 17 (College Station, TX, USA). All tests were 2 sided with a probability of a type 1 error set at .05. The study was deemed not regulated by our institutional review board.

## Results

Patient characteristics varied between practices with and without in-office dispensing, although most of these differences were small (Table 1). Specifically, men managed by practices without dispensing were older (82 vs 81 years, *P *=* *.003), of low socioeconomic status (40% vs 31%, *P *<* *.001), had 3 or more comorbid conditions (29% vs 27%, *P *=* *.001), and had a longer duration of androgen deprivation (35 months vs 33 months, *P *<* *.001). The distribution of men by race was balanced between practices with and without dispensing (Black men: 14% vs 13%, *P *=* *.10).

**Table 1.** Cohort characteristics, stratified by practices with and without in-office dispensing

Characteristics	With dispensing	Without dispensing	*P*
No.	6532	3811	
Age, mean (SD), y	81 (7)	82 (7)	.003
Socioeconomic status, No. (%)			<.001
Low	2012 (31)	1528 (40)	
Middle	2195 (34)	1253 (33)	
High	2325 (36)	1030 (27)	
Comorbidity, No. (%)			.001
0	2514 (38)	1475 (39)	
1	765 (20)	1299 (20)	
2	557 (15)	797 (12)	
≥3	1014 (27)	1922 (29)	
Race			.10
* *Non-Black men	5636 (86)	3332 (87)	
* *Black men	896 (14)	479 (13)	
Months of androgen deprivation therapy, mean (SD)	32 (24)	35 (26)	<.001

**Figure 1.**
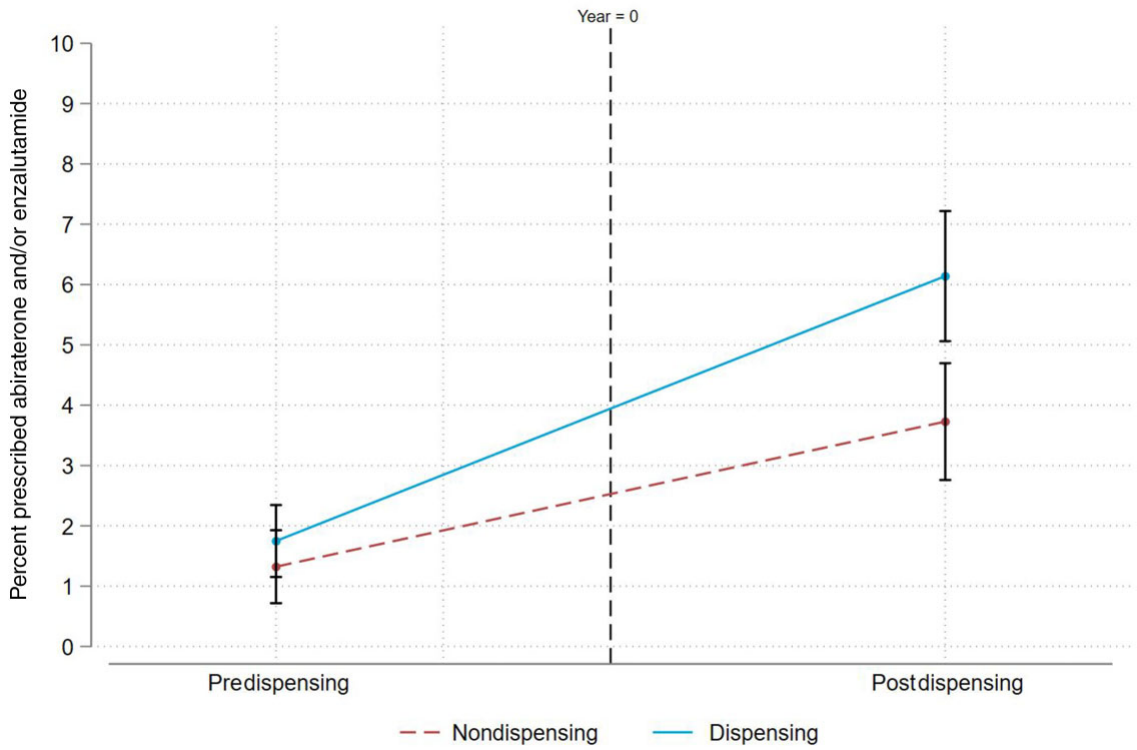
Adjusted probability of receiving a new prescription for abiraterone and/or enzalutamide before and after adoption of in-office dispensing.

Figure 1 illustrates the change in the percentage of men filling prescriptions for abiraterone and/or enzalutamide by practices with and without dispensing. Prior to adopting in-office dispensing, the percent of men filling prescriptions for abiraterone and/or enzalutamide managed by practices with dispensing was 1.7% (95% confidence interval [CI] = 1.2% to 2.3%) and 1.3% (95% CI = 0.7% to 1.9%) for those managed by practices without dispensing. After in-office dispensing adoption, the percent of men filling prescriptions managed by practices with dispensing increased to 6.1% (95% CI = 5.1% to 7.2%) compared with 3.7% (95% CI = 2.8% to 4.7%) for those managed by practices without dispensing. The increase in the postintervention period (difference-in-difference estimate) was 2% higher (95% CI = 0.6% to 3.4%) for practices with dispensing relative to practices without dispensing.

**Figure 2.**
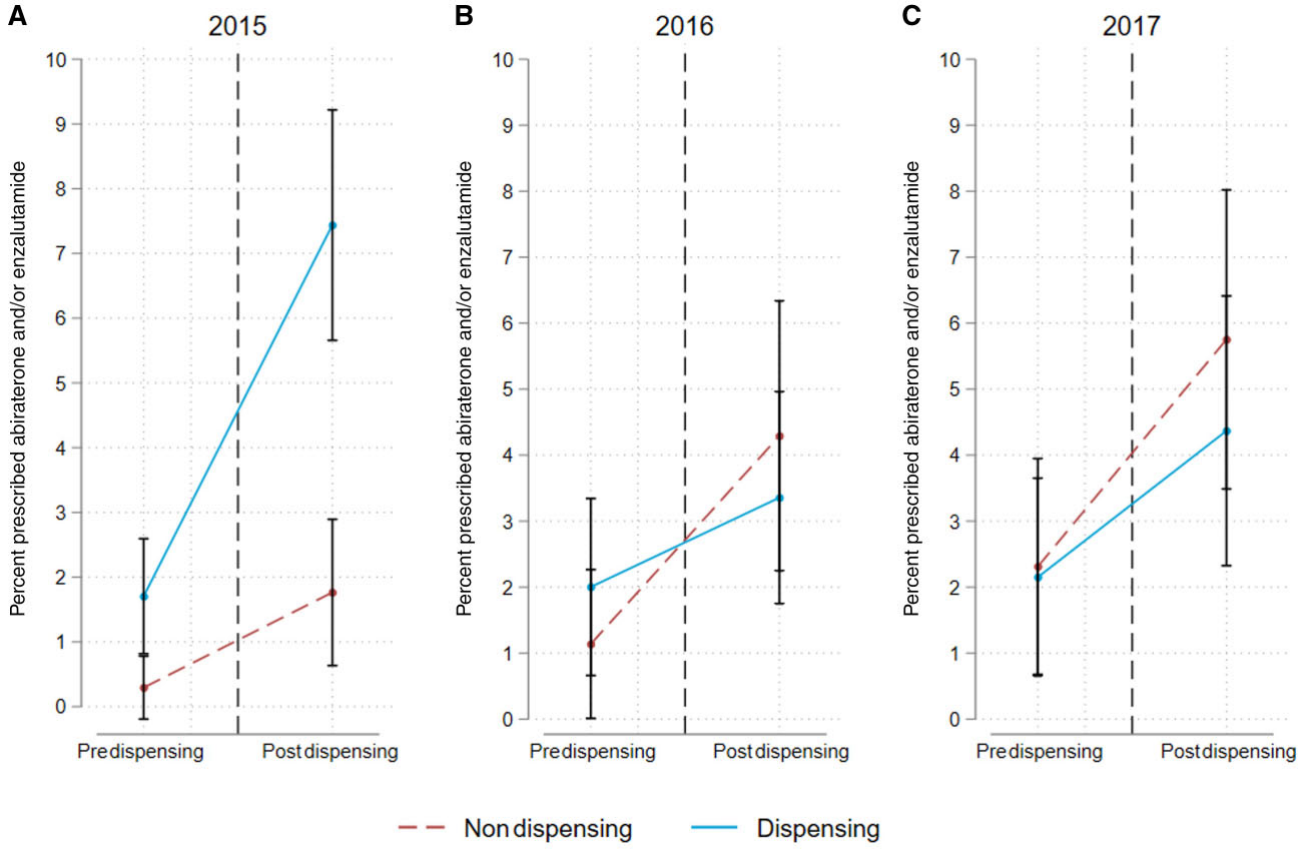
Adjusted probability of receiving a new prescription for abiraterone and/or enzalutamide before and after adoption of in-office dispensing, stratified by year of adoption: (**A)** 2015, (**B)** 2016, and (**C)** 2017.

Next, the effect of dispensing was assessed separately by year of adoption (Figure 2). For practices adopting dispensing in 2015, the percent of men filling prescriptions for abiraterone and/or enzalutamide in the pre-intervention period was 1.7% (95% CI = 0.8% to 2.6%) for those in practices with dispensing and 0.3% (95% CI = 0% to 0.7%) for those in practices without dispensing. Following adoption, the percent of men filling prescriptions increased to 7.4% (95% CI = 5.7% to 9.2%) for those in practices with dispensing compared with 1.8% (95% CI = 0.6% to 2.9%) for those in practices without dispensing. The increase in percent of men filling prescriptions in the postintervention period was 4.2% (95% CI = 2.3% to 6.2%) higher for practices with dispensing relative to practices without dispensing. Conversely, dispensing did not have a statistically significant effect for men managed by practices that adopted it in 2016 or 2017 (Table 2).

**Table 2.** Adjusted probabilities for receiving a new prescription for abiraterone and/or enzalutamide before and after dispensing adoption, among the entire cohort, by year of dispensing adoption and by race^a^

	With dispensing			Without dispensing			Δ,^b^ % (95% CI)	Δ Δ,^c^ % (95% CI)
	% (95% CI)			% (95% CI)				
	pre-intervention	Postintervention	*P*	pre-intervention	Postintervention	*P*		
Overall cohort	1.7 (1.2 to 2.3)	6.1 (5.1 to 7.2)	<.001	1.3 (0.7 to 1.9)	3.7 (2.8 to 4.7)	<.001	2.0 (0.6 to 3.4)	—
By year of dispensary opening								
2015	1.7 (0.8 to 2.6)	7.4 (5.7 to 9.2)	<.001	0.3 (0 to 0.7)	1.8 (0.6 to 2.9)	.01	4.2 (2.3 to 6.2)	—
2016	2.0 (0.6 to 3.3)	3.4 (1.7 to 5.0)	.14	1.1 (0 to 2.3)	4.3 (2.2 to 6.3)	.003	−1.8 (−4.5 to 1.0)	—
2017	2.2 (0.6 to 3.6)	4.3 (2.3 to 6.4)	.047	2.3 (0 to 3.9)	5.8 (3.5 to 8.0)	.009	−1.2 (−4.6 to 2.1)	—
By race								
Non-Black men	1.8 (1.2 to 2.4)	5.8 (4.7 to 6.9)	<.001	1.2 (0.6 to 1.8)	3.7 (2.7 to 4.7)	<.001	1.5 (0 to 3.0)	3.2 (−0.9 to 7.4)
Black men	1.4 (0.2 to 2.5)	8.0 (5.5 to 10.5)	<.001	2.1 (0.2 to 3.9)	4.0 (1.5 to 6.4)	.21	4.8 (0.8 to 8.7)	

a Models are adjusted for age, comorbidity, socioeconomic status, race, and months on androgen deprivation therapy. — = the difference value does not apply to those analyses; CI = confidence interval.

b Difference-in-difference estimate.

c Difference of difference-in-difference estimates between Black and non-Black men.

**Figure 3.**
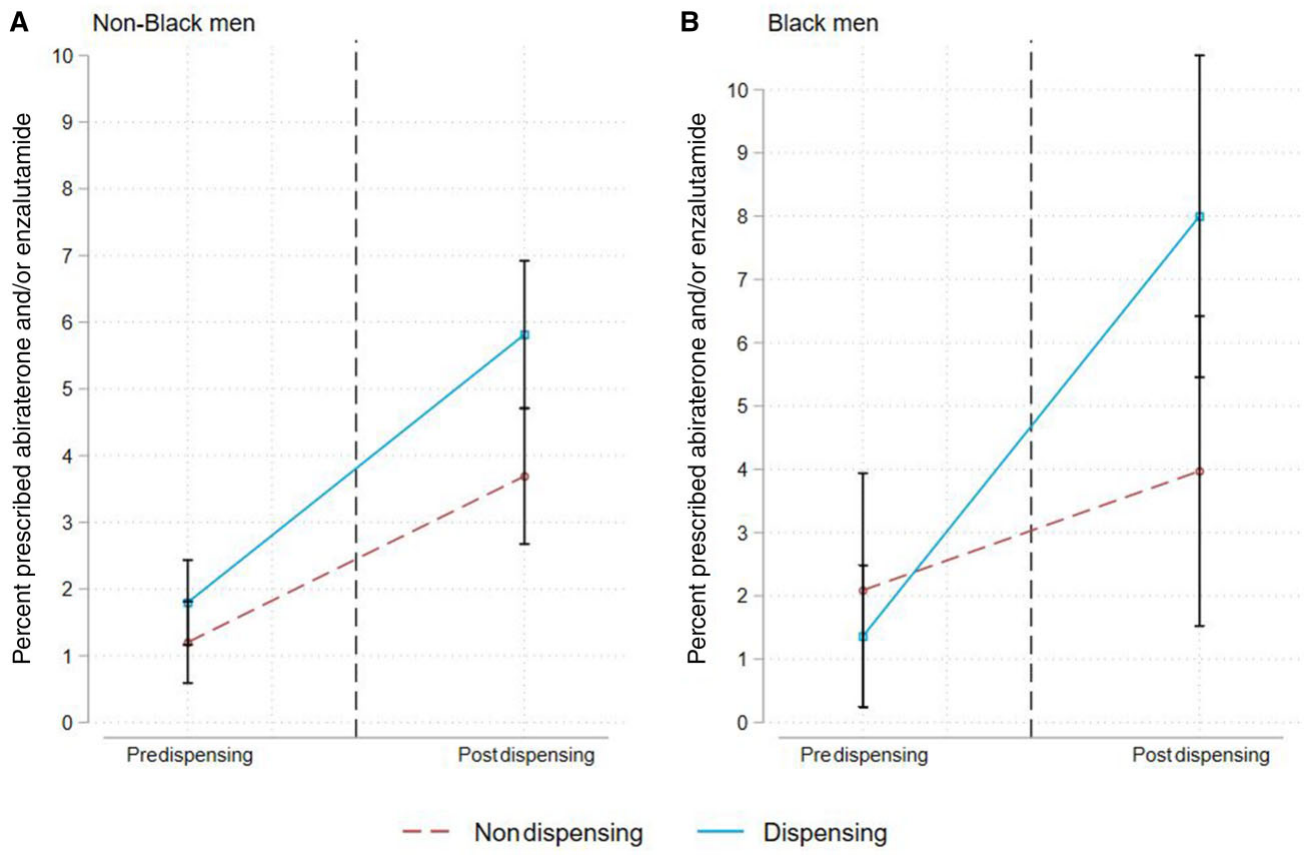
Adjusted probability of receiving a new prescription for abiraterone and/or enzalutamide before and after adoption of in-office dispensing, stratified by race: (**A)** non-Black men and (**B)** Black men.

Finally, we assessed whether the effect of dispensing differed between Black and non-Black men (Figure 3). Among non-Black men, the percent filling prescriptions for abiraterone and/or enzalutamide in the postintervention period was 1.5% (95% CI = 0.05% to 3.0%) higher for practices with dispensing relative to practices without dispensing. Among Black men, the percent of filling prescriptions in the postintervention period was 4.8% (95% CI = 0.8% to 8.7%) higher for practices with dispensing relative to practices without dispensing. The effect of in-office dispensing adoption among non-Black and Black men (ie, 1.5% vs 4.8%) was not statistically significantly different (3.2%, 95% CI = -0.9% to 7.4%).

## Discussion

Use of in-office dispensing among urology practices is becoming increasingly common. Importantly, this delivery model has the potential to increase use of novel targeted therapies for men with advanced prostate cancer. In this study of Medicare beneficiaries, we found that use of in-office dispensing resulted in an increase in percent of men filling a prescription for abiraterone and/or enzalutamide. This effect was primarily observed among practices initiating dispensing in 2015. Moreover, this effect did not vary by race, as Black and non-Black men managed by dispensing practices were similarly likely to fill a prescription for the targeted agents.

Men managed by urology practices with in-office dispensing were more likely to fill prescriptions for abiraterone and/or enzalutamide after dispensing adoption. However, this effect was most pronounced for men managed by practices adopting dispensing in 2015 and diminished for those in practices adopting dispensing in 2016 and 2017. This may be indicative of the evolving indications for targeted agents. For example, in 2015, abiraterone and enzalutamide were indicated for the late phase (ie, castrate resistant) of advanced prostate cancer, a disease state that was primarily managed by medical oncologists (20). Initiation of dispensing by urology practices may have expanded the pool of physicians through which men could obtain these medications thereby increasing access. Moreover, dispensing may facilitate prescribing among urologists by providing financial incentives, by increasing familiarity with the drugs by virtue of having the medications on-site, and by enabling continuity of care with their patients by offering treatments for advanced disease. However, increased familiarity of these agents by urologists—even those without in-office dispensing—and the expanding indications for their use over time (ie, in the castrate-sensitive phase of advanced prostate cancer) (1) may have contributed to the diminished effect of dispensing in the later years.

As new forms of care delivery become more common, it is important to assess their effect on preexisting disparities in health-care access and outcomes. Adoption of in-office dispensing by urology practices has the potential to increase access to targeted agents for advanced prostate cancer and thereby has the potential to mitigate existing disparities in treatment among Black men if the model is deployed equitably. Our findings suggest that the likelihood of receiving targeted agents following dispensing adoption did not differ between Black and non-Black men. This finding offers some reassurance that in-office dispensing is not deployed in a manner that exacerbates existing racial disparities in the treatment of advanced prostate cancer. However, it remains uncertain whether in-office dispensing has the potential to reduce or even eliminate these disparities. For example, prior work has demonstrated that Black men in the Veterans Affairs Health System were more likely to receive guideline-discordant treatment for advanced prostate cancer (6). Additionally, racial minorities, including Black men, are known to be at high risk for medication nonadherence in the context of prescription cancer medication (8). As such, strategic use of in-office dispensing may represent an avenue for mitigating disparities in cancer treatment. In fact, the observed effect of dispensing on receiving targeted agents for advanced prostate cancer was larger for Black men compared with non-Black men, although the difference between the 2 groups did not reach statistical significance possibly because of sample size limitations. Nonetheless, it is important to continue to monitor the impact of in-office dispensing on racial disparities and take measures to ensure that this delivery model does not inadvertently worsen them.

Our results must be interpreted in the context of several limitations. First, the inability to observe a statistically significant differential effect of in-office dispensing by race may be because of lack of power, given that Black men represent a minority of the Medicare population. Second, Medicare claims data do not contain information regarding disease stage or severity, and therefore it is not possible to ascertain the phase (castrate sensitive vs castrate resistant) or burden of advanced disease, which certainly dictates the type of treatment a patient receives and whether it is guideline concordant. Nonetheless, it is unlikely for disease stage or severity to be imbalanced when patients are aggregated in large groups, such as by practices with and without dispensing. Further, we adjusted for time on androgen deprivation therapy to serve as a surrogate for phase of advanced disease given that men on androgen deprivation therapy for longer periods of time are more likely to progress to the castrate-resistant phase (21). Third, the indications for use of targeted agents expanded during the study period, which may result in heterogeneous effects of dispensing depending on the year of adoption. Understanding this possibility, we decided a priori to analyze the data using a difference-in-difference design, both in aggregate and by fitting models for each year dispensing was adopted. From this approach, we were able to determine that the effect of dispensing was primarily observed for practices adopting dispensing in 2015. Finally, we excluded beneficiaries participating in multispecialty groups, which may also be likely to adopt in-office dispensing because of availability of resources and financial capital.

These limitations notwithstanding, our findings have important implications for patients and policy makers. In-office dispensing may accelerate prescriptions for oral targeted agents for men with advanced prostate cancer, which may have benefits and unintended consequences. For instance, dispensing these medications at the point of care may lead to improved access, timeliness, and adherence to specialty drugs for patients, including underrepresented minorities. However, financial incentives associated with physician dispensing, through a margin generated by a prescription fill, may promote utilization that undermines the value of this mode of delivery, such as treatment of patients who are unlikely to benefit or who are likely to experience adverse effects, leading to increased risk of financial burden and toxicity. Furthermore, dispensing may foster preferential use of therapies (eg, oral targeted drugs) over others that may be more effective in certain contexts (eg, docetaxel chemotherapy). Therefore, as dispensing becomes more common, there is a growing need to understand the potential benefits and harms of dispensing for patients.

In-office dispensing is a form of care delivery that is becoming increasingly common among urology practices. Men managed by practices that adopted in-office pharmacy were more likely to receive a new prescription for abiraterone and/or enzalutamide, although this effect was largely because of practices adopting dispensing in 2015. Further, the effect of dispensing was similar for Black and non-Black men.

## Data Availability

This study used Medicare claims data, provided by the Centers for Medicare & Medicaid Services (CMS) under license/by permission. Data may be shared on request to the corresponding author with permission of the CMS.
